# Phospholipids from herring roe improve plasma lipids and glucose tolerance in healthy, young adults

**DOI:** 10.1186/1476-511X-13-82

**Published:** 2014-05-17

**Authors:** Bodil Bjørndal, Elin Strand, Jennifer Gjerde, Pavol Bohov, Asbjørn Svardal, Bernd WK Diehl, Sheila M Innis, Alvin Berger, Rolf K Berge

**Affiliations:** 1 Department of Clinical Science, University of Bergen, Bergen N-5020, Norway; 2 Hormonlaboratoriet, Haukeland University Hospital, Bergen N-5021, Norway; 3 Department of Paediatrics, University of British Columbia, Vancouver, BC V5Z4H4, Canada; 4 Spectral Service AG, Köln D-50996, Germany; 5 Arctic Nutrition AS, Ørsta N-6155, Norway; 6 Department of Food Science & Nutrition, University of Minnesota, St. Paul, MN 55108-1038, USA; 7 Department of Heart Disease, Haukeland University Hospital, Bergen N-5021, Norway

**Keywords:** Herring roe, Phospholipids, Eicosapentaenoic acid, Docosahexaenoic acid, Omega-3 polyunsaturated fatty acids, Glycemic control, Choline, Acylcarnitines

## Abstract

**Background:**

Herring roe is an underutilized source of n-3 polyunsaturated fatty acids (PUFAs) for human consumption with high phospholipid (PL) content. Studies have shown that PL may improve bioavailability of n-3 PUFAs. Arctic Nutrition’s herring roe product MOPL™30 is a PL: docosahexaenoic acid (DHA)-rich fish oil mixture, with a DHA:eicosapentaenoic acid (EPA) ratio of about 3:1, which is also rich in choline. In this pilot study, we determined if MOPL30 could favorably affect plasma lipid parameters and glucose tolerance in healthy young adults.

**Methods:**

Twenty female and one male adults, between 22 and 26 years of age, participated in the study. Participants took encapsulated MOPL30, 2.4 g/d EPA + DHA, for 14 days, and completed a three-day weighed food record before and during the capsule intake. Plasma lipids and their fatty acid (FA) composition, plasma and red blood cell (RBC) phosphatidylcholine (PC) FA composition, acylcarnitines, choline, betaine and insulin were measured before and after supplementation (n = 21), and one and four weeks after discontinuation of supplementation (n = 14). An oral glucose tolerance test was performed before and after supplementation.

**Results:**

Fasting plasma triacylglycerol and non-esterified fatty acids decreased and HDL-cholesterol increased after 14 days of MOPL30 intake (p < 0.05). The dietary records showed that PUFA intake prior to and during capsule intake was not different. Fasting plasma glucose was unchanged from before to after supplementation. However, during oral glucose tolerance testing, blood glucose at both 10 and 120 min was significantly lower after supplementation with MOPL30 compared to baseline measurements. Plasma free choline and betaine were increased, and the n-6/n-3 polyunsaturated (PUFA) ratio in plasma and RBC PC were decreased post-supplementation. Four weeks after discontinuation of MOPL30, most parameters had returned to baseline, but a delayed effect was observed on n-6 PUFAs.

**Conclusions:**

Herring roe rich in PL improved the plasma lipid profile and glycemic control in young adults with an overall healthy lifestyle.

## Background

The health benefits of a higher fish intake, thereby increasing the intake of n-3 long-chain polyunsaturated fatty acids (PUFAs) and reducing the n-6 PUFA/n-3 PUFA ratio, has been documented in several studies [1]. Cardioprotective effects of n-3 PUFAs, in particular eicosapentaenoic acid (EPA) and docosahexaenoic acid (DHA), have been attributed to reduction in fasting triacylglycerol (TAG), blood pressure lowering, anti-inflammatory and antiarrhythmic effects, improved insulin sensitivity and vascular endothelial function, and reduced thrombotic tendency [2]. The efficacy of n-3 PUFAs in the prevention of heart disease has been challenged in recent meta-studies, but it is important to note that newer studies could be hampered by a higher general intake of n-3 PUFAs and improved treatment protocols for heart patients [3]. The current recommended intake is 250 mg/day EPA + DHA for the general population, and 300 mg/day for pregnant women (European Food Safety Authority (EFSA)). The American Heart Association (AHA) recommends 1 g/day EPA + DHA for patients with cardiovascular disease (CVD). Combined with the increased focus on n-3 PUFA intake in the media, this has led to a large n-3 PUFA supplement market dominated by fish oil from sardines. However, the need for new sources of high-quality EPA and DHA is increasing.

Immature roe from spring-spawning Norwegian herring is an underutilized source of n-3 PUFA-rich phospholipids (PLs). Of the approximately 600,000-ton herring caught in Norway each year, only a small percentage of herring roe is used for human consumption. The product MOPL30 (Arctic Nutrition) from herring roe contains about 45% n-3 PUFA (mg/g product basis), with a DHA:EPA ratio of approximately 3:1. In addition, 30% of the lipids are PL of which most (75%) in the form of phosphatidylcholine (PC). Thus herring roe provides choline, an important nutrient involved in many biochemical pathways.

The bioactivity of n-3 PUFAs may be influenced by the lipid structures in which they are incorporated. PLs and free fatty acids have increased bioavailability compared to TAG and ethyl ester forms of n-3 PUFAs, respectively [4,5]. PL from krill have been shown to influence gene expression more than TAG from fish oil in mice, at a similar dose of EPA and DHA [6]. In particular, genes involved in glucose and lipid metabolism were more affected by PL than TAG [6,7]. A recent study comparing the bioavailability of PL and TAG in the form of krill oil and fish oil in healthy subjects demonstrated increased levels of EPA and DHA in plasma and red blood cells (RBC) after four weeks of krill oil intake compared to fish oil [8]. In addition, animal studies have shown a more efficient reduction in plasma lipid levels with PL compared to TAG intervention [9]. Herring roe oil supplementation has shown promising results in animal studies, including reduction in plasma lipids and inflammatory parameters, and improved insulin sensitivity [10,11].

The aim of this study was to determine the effect of n-3 PUFA when given in PL-rich herring roe on blood lipids and glucose tolerance in healthy subjects with a balanced diet, and to determine uptake of n-3 PUFAs into red blood cells as a measure of bioavailability. Follow-up samples were included to determine how long the n-3 PUFA remained in circulation after discontinuation of supplementation (washout effects).

## Results

### Characteristics of the study population

The study included twenty-one young, healthy individuals aged 20 to 26 years, with a mean ± SD body mass index of 21.2 ± 2.8, and range between 15.1 and 26.7. Fourteen of the participants completed a three day weighed food record during the two weeks prior to supplementation, and again during the two weeks intervention. The energy % from PUFA in the habitual diet was (mean ± SD) 7.5 ± 2.8 before and 6.7 ± 2.0 during the intervention (p = 0.264, n = 14), indicating that the dietary intake of PUFA remained unchanged during the study period. Furthermore, there was no change in dietary intake of total fat, saturated fatty acids (FA), monounsaturated FA, protein or carbohydrate (data not shown).

The capsules were taken during a meal, and there were no reports of reflux or unpleasant taste following capsule intake.

### MOPL30 supplementation affected plasma lipid levels

**Figure 1:**
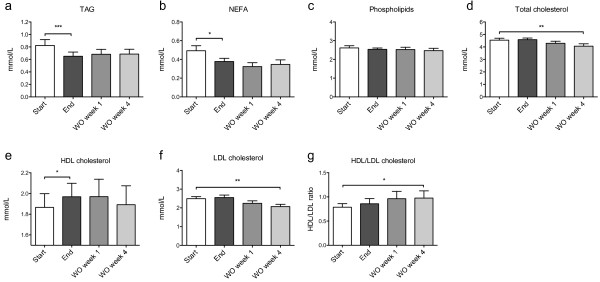
**Plasma lipid levels in response to MOPL30.** Levels of triacylglycerol (TAG, **a)**, non-esterified fatty acids (NEFA, **b)**, phospholipids **(c)**, total cholesterol **(d)**, high-density lipoprotein (HDL) cholesterol **(e)**, low-density lipoprotein (LDL) cholesterol **(f)**, and the HDL/LDL cholesterol ratio **(g)** in fasted plasma samples taken at baseline (start), after 14 days of supplement (end), and one week (wash out (WO) week 1) and four weeks (WO week 4) after discontinuation of supplement. Values are given as means with standard deviations, and significant changes between start and end (n = 21) and start and WO (n = 13) are indicated (*p < 0.05, **p < 0.01, ***p < 0.001).

Plasma lipid levels were measured at baseline (start) and after (end) two weeks of MOPL30 supplementation. In addition, washout (WO) samples were taken one and four weeks after the final day of capsule intake. The TAG level was significantly reduced in the end samples (21% reduction). However, the reduction at WO week one (p = 0.10) and four (p = 0.30) compared to start was not significant (Figure 1a). Non-esterified FAs (NEFAs) were also significantly reduced after two weeks of MOPL30 intake (23.3%), and remained unchanged, however insignificantly, one (34.3%, p = 0.17) and four weeks (29.8%, p = 0,24) after discontinued supplementation (Figure 1b). Despite a high PL content in the supplement, plasma PL levels were not altered (Figure 1c).

The total cholesterol level in plasma was not changed by MOPL30 intake (Figure 1d). HDL-cholesterol, however, increased by 5.5% at end compared to start (Figure 1e), while LDL-cholesterol was unchanged (Figure 1f). This led to a trend towards an increased HDL/LDL-cholesterol after MOPL30 intake (9.0%; Figure 1g). Surprisingly, while plasma HDL-cholesterol returned to initial values after four weeks of WO, total- and LDL-cholesterol was significantly reduced at 4 week WO compared to start samples (10.6% reduction in WO week 4 vs. start).

### Fatty acid composition in plasma and red blood cells

Total fatty acid composition was measured in plasma before and after two weeks of supplement. Total n-3 PUFAs in plasma were increased 1.6 fold, mainly due to a 2.2 fold increase in EPA, and a 1.5 fold increase in DHA (Table 1). One week after the end of supplementation, EPA and DHA had decreased by 44% and 24%, respectively, compared to the end values. By four weeks of WO, all plasma n-3 PUFAs had returned to start levels. Concomitant with the increase in EPA and DHA during the 2 weeks supplementation, n-6 PUFAs showed a decrease of 8.2%. Interestingly, n-6 PUFAs, in particular arachidonic acid (AA) did not return to start levels as quickly as EPA and DHA after discontinuation of supplementation.

**Table 1:** Plasma fatty acids during the study

**μg FA/ml plasma**	**Start**	**End**	**WO 1 week**	**WO 4 weeks**
∑ FAs	2999 ± 605	2811 ± 449*	2767 ± 409*	2677 ± 443**
∑ SFAs	956.4 ± 219.2	881.5 ± 151.1*	875.4 ± 154.8	708.6 ± 207.1**
C16:0	651.9 ± 172.0	589.5 ± 121.6**	582.0 ± 119.6	558.2 ± 121.6**
∑ MUFAs	708.6 ± 207.1	573.8 ± 124.8***	613.4 ± 132.7*	583.4 ± 140.5*
C16:1n-7	39.8 ± 19.2	30.8 ± 11.6*	34.6 ± 20.3	34.1 ± 17.6
C16:1n-9	6.91 ± 2.85	6.10 ± 2.08*	6.63 ± 2.80	6.90 ± 2.75*
C18:1*n-*7	45.2 ± 15.4	36.5 ± 10.6**	36.1 ± 6.6*	36.4 ± 9.3
C18:1*n-*9	568.2 ± 171.9	454.0 ± 101.0***	491.9 ± 112.1*	463.4 ± 111.1*
∑ *n-*9 PUFAs	3.10 ± 1.29	2.24 ± 0.48**	2.75 ± 1.38	2.55 ± 1.01**
C20:3*n-*9 (MA)	3.10 ± 1.29	2.24 ± 0.48**	2.75 ± 1.38	2.55 ± 1.01**
∑ *n-*6 PUFAs	1123 ± 190	1031 ± 180*	1036 ± 148*	1049 ± 141*
C18:2n-6	862.7 ± 147.9	806.2 ± 153.3	814.5 ± 110.1	821.0 ± 93.7
C20:2*n-*6	6.26 ± 2.43	4.91 ± 1.76***	5.52 ± 1.64	5.90 ± 2.08
C18:3n-6	7.75 ± 5.00	4.33 ± 2.41***	6.96 ± 4.075	7.16 ± 6.84
C20:3*n-*6	40.5 ± 17.3	27.7 ± 9.93***	33.3 ± 9.78*	35.4 ± 13.9
C20:4*n-*6 (AA)	197.8 ± 51.6	182.3 ± 41.1*	170.3 ± 41.6***	173.0 ± 50.9*
C22:4*n-*6	4.33 ± 1.58	2.92 ± 0.71***	3.32 ± 0.92**	3.70 ± 1.56**
∑ *n-*3 PUFAs	200.8 ± 64.1	315.6 ± 62.8***	232.7 ± 62.9	197.0 ± 74.0
C18:3n-3	19.8 ± 8.17	16.6 ± 6.06*	20.0 ± 8.85	19.0 ± 8.0
C20:5*n-*3 (EPA)	49.4 ± 27.1	109.7 ± 37.1***	61.7 ± 31.7	47.3 ± 32.7
C22:5*n-*3 (DPA)	17.6 ± 5.36	18.4 ± 4.27	18.0 ± 4.22	17.0 ± 5.43
C22:6*n-*3 (DHA)	108.1 ± 30.4	166.1 ± 30.5***	127.0 ± 29.5**	108.4 ± 35.2
∑ *n-*6:∑ *n-*3 ratio	6.10 ± 1.81	3.37 ± 0.82***	4.75 ± 1.42*	5.88 ± 1.72

*Abbreviations*: *AA* arachidonic acid, *DHA* docosahexaenoic acid; *EPA* eicosapentaenoic acid; *FA* fatty acid; *MA* mead acid; *MUFA* unsaturated fatty acids; *PUFA* polyunsaturated fatty acids; *SFA* saturated fatty acids; *WO*, wash out.

Data are means ± SD. Significantly different end and start values (n = 21) and WO and start values (n = 13) are indicated (*p < 0.05, **p < 0.01, ***p < 0.001).

Rather AA remained lower than start values in the four-week WO samples. Although mead acid was low in start samples, as expected with a high habitual PUFA intake, it still showed a significant decrease with the MOPL30 supplement (27.7% reduction). The total fatty acid content in plasma was significantly reduced after two weeks of supplement, consistent with the reduced TAG and NEFA. The n-6/n-3 PUFA ratio was within recommendations in the start samples, consistent with the participants’ balanced intake of fatty acids (Table 1). The n-6/n-3 ratio was further decreased by 44.8% post-supplementation, but had returned to start values at WO week 4.

As most of the EPA and DHA in MOPL30 are in the form of PC, the fatty acid composition of PC was measured both in plasma and in red blood cells. The findings in plasma PC paralleled the findings in total plasma FAs, with a 2.1 fold increase in EPA and a 1.6 increase in DHA post-supplementation, resulting in a 45.2% decrease in the n-6/n-3 PUFA ratio (Table 2). At WO week 1, EPA and DHA were reduced by 39.3% and 21.9%, respectively, relative to end values. Incorporation of EPA and DHA into RBC membranes is believed to better indicate long term storage than plasma levels [12]. We found a 2.1 fold increase in EPA and a 1.4 fold increase in DHA post-supplement, similar to results in plasma, resulting in a 1.7 fold increase in the RBC PC omega-3 index and a 40.6% reduction in the n-6/n-3 PUFA ratio (Table 3). AA in the plasma PC and RBC PC was not significantly altered by MOPL30, although it was significantly reduced in plasma total fatty acids (Tables 1, 2 and 3). The WO effect on the RBC PC EPA was similar to that in the plasma PC after one week (39% reduction relative to end values), while the decrease in the RBC PC DHA was lower (11% reduction relative to end values).

**Table 2:** Plasma phosphatidylcholine fatty acids during the study

**Fatty acids, wt%**	**Start**	**End**	**WO 1 week**	**WO 4 weeks**
C16:0	27.1 ± 3.11	26.8 ± 3.07	26.6 ± 3.73	27.2 ± 3.54
C16:1n-7	0.48 ± 0.14	0.46 ± 0.10	0.48 ± 0.25	0.79 ± 0.82
C18:1*n-*7	1.54 ± 0.20	1.52 ± 0.25	1.50 ± 0.25	1.41 ± 0.24
C18:1*n-*9	10.8 ± 1.66	9.60 ± 1.14**	10.9 ± 1.92	10.6 ± 2.03
C20:3*n-*9 (MA)	0.12 ± 0.04	0.08 ± 0.02***	0.11 ± 0.06	0.11 ± 0.04*
C18:2n-6	23.8 ± 2.73	21.9 ± 2.75*	23.6 ± 2.16	24.2 ± 3.02
C20:2*n-*6	0.53 ± 0.10	0.47 ± 0.07***	0.50 ± 0.10	0.51 ± 0.10
C20:3*n-*6	2.58 ± 0.78	1.87 ± 0.55***	2.26 ± 0.54	2.43 ± 0.68
C20:4*n-*6 (AA)	8.07 ± 1.31	7.65 ± 1.34	7.16 ± 1.49*	7.34 ± 1.53
C22:4*n-*6	0.26 ± 0.20	0.17 ± 0.03*	0.18 ± 0.04**	0.20 ± 0.07
C18:3n-3	0.25 ± 0.08	0.20 ± 0.06**	0.30 ± 0.15	0.30 ± 0.02
C20:5*n-*3 (EPA)	1.90 ± 1.01	3.97 ± 1.43***	2.41 ± 1.30	1.84 ± 1.02
C22:5*n-*3 (DPA)	0.77 ± 0.17	0.85 ± 0.20	0.82 ± 0.17	0.76 ± 0.15
C22:6*n-*3 (DHA)	4.20 ± 0.87	6.52 ± 1.10***	5.09 ± 1.03**	4.25 ± 0.67
∑ *n-*6:∑ *n-*3 ratio	5.29 ± 1.41	2.90 ± 0.68***	4.15 ± 1.13	5.12 ± 1.23

*Abbreviations*: *AA* arachidonic acid, *DHA* docosahexaenoic acid; *EPA* eicosapentaenoic acid; *FA* fatty acid; *MA* mead acid; *MUFA* unsaturated fatty acids; *PUFA* polyunsaturated fatty acids; *SFA* saturated fatty acids; *WO* wash out.

Data are means ± SD. Significantly different end and start values (n = 21) and WO and start values (n = 13) are indicated (*p < 0.05, **p < 0.01, ***p < 0.001).

**Table 3:** Red blood cell phosphatidylcholine fatty acids during the study

**Fatty acids, wt%**	**Start**	**End**	**WO 1 week**	**WO 4 weeks**
C16:0	32.2 ± 3.62	31.3 ± 3.43	31.6 ± 3.46	31.6 ± 3.87
C16:1n-7	0.33 ± 0.15	0.24 ± 0.05**	0.30 ± 0.14	0.29 ± 0.12
C18:1*n-*7	1.65 ± 0.19	1.67 ± 0.18	1.60 ± 0.17	1.62 ± 0.15
C18:1*n-*9	16.2 ± 1.53	15.8 ± 0.98*	16.1 ± 1.00	15.8 ± 1.20
C20:3*n-*9 (MA)	0.06 ± 0.02	0.05 ± 0.02	0.05 ± 0.02*	0.05 ± 0.02
C18:2n-6	20.0 ± 1.65	18.8 ± 1.80***	20.2 ± 1.55	20.96 ± 1.83
C20:2*n-*6	0.32 ± 0.10	0.34 ± 0.06	0.32 ± 0.05	0.34 ± 0.07
C20:3*n-*6	1.65 ± 0.06	1.34 ± 0.38***	1.35 ± 0.32***	1.49 ± 0.39
C20:4*n-*6 (AA)	5.33 ± 0.90	5.18 ± 0.86	4.97 ± 0.91*	4.48 ± 1.48
C22:4*n-*6	0.22 ± 0.09	0.22 ± 0.06	0.23 ± 0.04	0.21 ± 0.07
C18:3n-3	0.18 ± 0.07	0.23 ± 0.28	0.18 ± 0.07	0.17 ± 0.05
C20:5*n-*3 (EPA)	1.10 ± 0.47	2.35 ± 0.85***	1.43 ± 0.57	1.08 ± 0.54
C22:5*n-*3	0.53 ± 0.10	0.56 ± 0.13	0.56 ± 0.11	0.55 ± 0.11
C22:6*n-*3 (DHA)	2.43 ± 0.52	3.48 ± 0.60***	3.10 ± 0.63**	2.58 ± 0.52
∑ *n-*6:∑ *n-*3 ratio	6.85 ± 1.64	4.07 ± 0.80***	5.37 ± 1.12**	6.58 ± 1.40
Omega-3 index	3.53 ± 0.88	5.83 ± 1.26***	4.52 ± 0.94*	3.65 ± 0.84

*Abbreviations*: *AA* arachidonic acid, *DHA* docosahexaenoic acid; *EPA* eicosapentaenoic acid; *FA* fatty acid; *MA* mead acid; *MUFA* unsaturated fatty acids; *PUFA* polyunsaturated fatty acids; *SFA* saturated fatty acids; *WO* wash out.

Data are means ± SD. Significantly different end and start values (n = 21) and WO and start values (n = 13) are indicated (*p < 0.05, **p < 0.01, ***p < 0.001).

### Plasma choline increased with MOPL30

**Figure 2:**
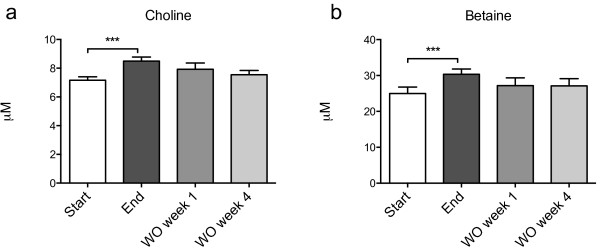
**Plasma choline and betaine levels in response to MOPL30.** Levels of choline **(a)** and betaine **(b)** in fasted plasma samples taken at baseline (start), after 14 days of supplement (end), and one week (wash out (WO) week 1) and four weeks (WO week 4) after discontinuation of supplement. Values are given as means with standard deviations, and significant changes between start and end (n = 21) and start and WO (n = 13) are indicated (*p < 0.05, **p < 0.01, ***p < 0.001).

**Figure 3:**
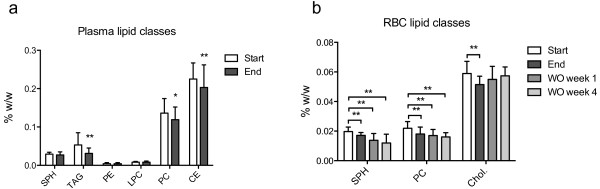
**Lipid classes in plasma and red blood cells (RBC) in response to MOPL30.** Comparison of the lipid classes sphingomyelin (SPH), triacylglycerol (TAG), phosphatidyl ethanolamine (PE), lysophosphatidylethanolamine (LPC), phosphatidyl choline (PC), and cholesterol esters (CE) at baseline (start) and after 14 days of supplement (end) in fasting plasma samples **(a)**. Comparison of the lipid classes SPH, TAG, and cholesterol (Chol.) at start, end, and one week (wash out (WO) week 1) and four weeks (WO week 4) after discontinuation of supplement in RBC **(b)**. Analysis was done by ^31^P NMR. Values are given as means (%w/w = g/100 g plasma) with standard deviations, and significant changes between start and end (n = 21) and start and WO (n = 13) are indicated (*p < 0.05, **p < 0.01).

Both choline and its metabolite betaine increased in plasma after two weeks of MOPL30 supplement (Figure 2). Although the intake of PC was increased during supplementation, total plasma- and RBC PCs were reduced post-supplementation, as measured by ^31^P nuclear magnetic resonance (NMR) (Figure 3a and b). However, a higher proportion of EPA and DHA-containing PCs were observed in both plasma and RBC (Tables 2 and 3). Plasma TAG was reduced, while no change was seen in cholesterol esters (Figure 3a). Total cholesterol, measured by ^1^H NMR, was not influenced by two weeks of supplement (mean ± SD start vs end; 0.95 ± 0.18 mmol/L vs 0.88 ± 0.019 mmol/L, p = 0.096). The plasma TAG level measured with NMR was similar to the plasma TAG-level measured by enzymatic-analysis (Figure 1). Sphingomyelin (SPH), PC, and cholesterol were reduced in RBC by two weeks of MOPL30 treatment (Figure 3b). Interestingly, while cholesterol returned to start levels after four weeks WO, SPH and PC remained lower than start values.

### Plasma carnitine and acylcarnitine levels

**Figure 4:**
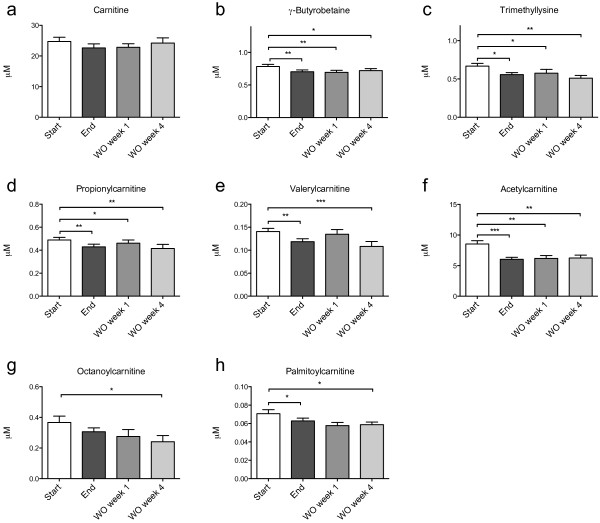
**Plasma carnitine and acylcarnitines in response to MOPL30.** Levels of carnitine **(a)** and the carnitine precursors gamma-butyrobetaine **(b)**, trimethylysine **(c)**, even-chain acylcarnitines propionylcarnitine **(d)**, and isovaleryl/valerylcarnitine **(e)**, and odd-chain acylcarnitines acetylcarnitine **(f)**, octanoylcarnitine **(g)**, and palmitoylcarnitine **(h)** in fasted plasma samples taken at baseline (start), after 14 days of supplement (end), and one week (wash out (WO) week 1) and four weeks (WO week 4) after discontinuation of supplement. Number of carbons in the acyl-chains are indicated by C2-16. Values are given as means with standard deviations, and significant changes between start and end (n = 21) and start and WO (n = 13) are indicated (*p < 0.05, **p < 0.01, ***p < 0.001).

Carnitine is essential in the transport of long-chain fatty acids across the mitochondrial membranes. High serum levels of long- and medium-chain plasma acylcarnitines are linked to increased risk of disease progression in patients with cardiac disease, and may indicate defects in mitochondrial function [13]. In the current study with healthy individuals, carnitine was insignificantly reduced (p = 0.054), while its precursors γ-butyrobetaine and trimethyllysine were reduced by MOPL30 (Figure 4a-c). In addition, all measured plasma acylcarnitines except the medium-chain octanoylcarnitine (8-carbon, p = 0.103), were significantly reduced by MOPL30 (Figure 4d-h). The largest reduction (29% compared to start) was seen for the short-chain acetylcarnitine (2-carbon). The effect on γ-butyrobetaine, trimethyllysine and all acylcarnitines remained significantly reduced compared to start in both the one- and four week WO samples, indicating a possible prolonged effect of the supplement on these parameters.

### Oral glucose tolerance test

**Figure 5:**
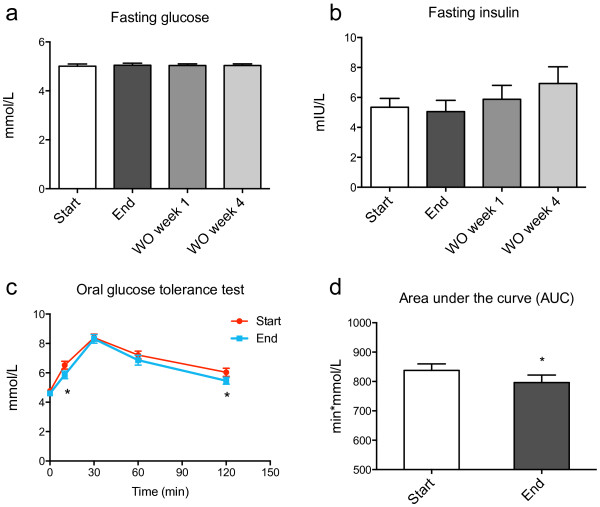
**Fasting glucose and insulin levels, and oral glucose tolerance.** Levels of glucose **(a)**, insulin **(b)**, in fasted plasma and serum samples, respectively, taken at baseline (start), after 14 days of supplement (end), and one week (wash out (WO) week 1) and four weeks (WO week 4) after discontinuation of supplement. Significant changes between start and end (n = 21) and start and WO (n = 13) are indicated by P-values. Oral glucose toleranse was measured at start and end of the experiment, blood glucose levels at baseline, 10, 30, 60, and 120 minutes after glucose ingestion are given **(c)**. The area under the curve was calculated at start and end **(d)**. All values are given as means with standard deviations, and significant changes between start and end (n = 21) are indicated by *p < 0.05.

Since previous studies in animals have indicated improved glucose metabolism after herring roe diets, we investigated the effect of two weeks MOPL30 supplement on glucose tolerance in healthy individuals. No change was observed in fasting insulin and glucose before and after the supplementation period (Figure 5a and b). However, the blood glucose level in response to a 75 g oral dose of glucose was reduced both at 10 and 120 minutes post-ingestion after the intervention (Figure 5c). Although all participants were individuals with normal glucose sensitivity according to their glucose tolerance test results, the area under the curve was significantly decreased, suggesting improved glucose response (Figure 5d).

## Discussion

There is an increased demand for new sources of high-quality n-3 PUFAs for human consumption, and PC-rich lipids from herring roe is a promising product in this regard. Supplementation with DHA- and EPA-rich PC may have additional benefits compared to DHA- and EPA-rich TAG both due to PL being more easily incorporated into cellular membranes, as well as being a source of the essential nutrient choline. In this two-week intervention in young healthy adults with high habitual fish intakes, there was a rapid increase in EPA and DHA in RBC PC, plasma FA and plasma PC. Furthermore, there was a corresponding improved lipid status, including a decrease in plasma TAG and NEFA, and increased HDL-cholesterol, choline, and betaine. These findings demonstrate that MOPL30 had significant biological effects in healthy subjects.

The participants were given 2350 mg EPA and DHA daily, comparable to doses utilized in patients with CVD or metabolic syndrome to achieve a TAG-lowering effect. In line with this, plasma TAG was reduced and HDL-cholesterol increased after only two weeks of treatment. This indicates a high bioavailability of MOPL30 and a subsequent rapid effect on lipid metabolism. It has been shown that the preferred lipid form for transport of DHA to RBC is lyso-PC, which is rapidly converted to PC [14,15]. This could mean that DHA-rich PC may be preferred for uptake in RBC and putatively in brain. Incorporation of EPA from fish oil into RBC membranes has been shown to reach a steady state after 180 days [12]. Notably, we observed an increase in EPA and DHA in RBC PC similar to that of plasma after only 14 days. In mice, we recently showed that similar amounts of EPA and DHA in liver PL were achieved with krill oil and a two-fold higher dose of EPA and DHA from fish oil, indicating higher bioavailability of EPA and DHA from the PL-source krill oil [16]. Importantly, in a recent study in which subjects were supplemented with 600 mg EPA and DHA from either fish- or krill oil, the omega-3 index was significantly higher in subjects receiving krill oil than in those given fish oil [8]. However, no effect on plasma TAG was observed at 600 mg/day EPA + DHA. A recent study in adults with high TAG levels showed that a dose of 0.5-2 g/day krill oil for 12 weeks significantly reduced TAG [17].

Although the intake of DHA exceeded that of EPA by 2.8 fold, the increase in EPA was higher than DHA in the PC-fraction of both plasma and red blood cells after two weeks of MOPL30. Studies have shown that the DHA level in lipid pools has a less steep dose–response curve than EPA, which is easy to influence by supplementation [18], and our results confirm this. This can partly be due to more efficient liberation of DHA from chylomicrons [19], leaving more EPA in chylomicron remnants and hereby making more EPA available for PL synthesis in liver. In addition, retro-conversion of DHA to EPA is dependent on peroxisomal β-oxidation, and is reported to be at approximately 10% in humans [20,21]. Hansen et al. showed that 4 g supplement of pure EPA for 5 weeks led to a 6.2 fold increase in EPA in plasma phospholipids, and no change in DHA [18]. In contrast, the same intervention using a pure DHA supplement led to a 1.9 fold increase in DHA and a 1.7 fold increase in EPA. This demonstrates the importance of supplementation with DHA and not only EPA. Despite a dose of 132 mg DPA/day, and the possibility of formation of DPA from EPA, DPA levels were not influenced by two weeks of MOPL30. In line with this, a decrease in DPA after uptake of EPA or DHA has been reported in long-term studies [22,23].

Indications of differential effects of n-3 PUFA in PL and TAG form have also been found at the gene level in several animal studies [6,7], including genes involved in glucose metabolism. A recent study reported that EPA and DHA supplements may improve insulin sensitivity in young obese individuals [24]. While some meta-studies have failed to show an effect between n-3 PUFA intake and incident type 2 diabetes mellitus (T2DM) [25,26], others indicate a reduced risk of T2DM with increased intake of PUFAs [27,28]. Based on findings in humans and from recent animal studies, PL supplements could be expected to have potent effects on glucose metabolism. The small, but significant, improvement in glucose response in healthy individuals after only two weeks of intervention suggests a potential for the use of MOPL30 in insulin resistant individuals, or patients with diabetes.

The conditionally essential nutrient choline is a quaternary amine, and is mainly utilized for the synthesis of PC and sphingomyelin, as well as lysophosphatidylcholine. In addition, choline can be oxidized to betaine, which is involved in the remethylation of homocysteine to methionine in the one-carbon cycle [29]. Finally, in the neurons choline is a precursor for the important neurotransmittor and vasodilator acetylcholine [30-32], and increased intake has been connected with improved cognition, learning and memory [33-35]. Betaine holds an important role in the liver, and has a potential therapeutic use in the treatment of fatty liver disease as well as homocysteinemia, a risk factor for CVD [36,37]. In addition, some studies have demonstrated that betaine supplements improve muscle performance [38]. PC biosynthesis is required for VLDL production, both through the CDP-choline (Kennedy) pathway and the phosphatidylethanolamine N-methyltransferase (PEMT) pathway. In general, a balanced diet will provide sufficient amounts of choline, but groups which may benefit from choline supplementation are pregnant and lactating women, infants, and cirrhosis patients [39]. Thus, a PC supplement can both reduce the need for methyl-donors for PC synthesis, and supply betaine for homocysteine remethylation, which will be beneficial in situations where methyl donors are limited [40]. Interestingly, while MOPL30 supplementation led to increased EPA and DHA-rich PC in plasma and RBC, the total level of PC decreased, with a concomitant increase in plasma free choline. This may indicate a higher level of PC degradation as a result of increased dietary intake, ensuring maintenance of the strictly regulated choline balance in the human body [41]. We were unable to measure acetylcholine in plasma due to its short half-life, however, both the one-carbon cycle/remethylation process and the production of acetylcholine may potentially have been stimulated by increased choline levels. DHA has been demonstrated to increase synaptic transmission in mammalian brain cells, and this effect was potentiated by phosphatidylcholine [42]. Thus, MOPL30 may have beneficial effects on cognitive function. It would be valuable to measure plasma choline acetyltransferase activity in future clinical trials to verify if acetylcholine production is stimulated by MOPL30 in humans.

As high plasma levels of long- and medium-chain acylcarnitines are linked to increased heart failure in CVD patients, they have been put forward as potential biomarkers of cardiovascular risk [13]. Incomplete β-oxidation, impaired substrate switching, and dysregulation of mitochondria during insulin resistance can cause elevated levels of intermediate oxidation products, and this can be reflected in plasma acylcarnitine levels [43,44]. Thus, it is of interest to establish whether dietary intervention with n-3 PUFAs affect these plasma parameters in healthy adults, as EPA and DHA are known to stimulate mitochondrial β-oxidation. We observed a reduction in all acylcarnitines after a two-week intervention with MOPL30, including the risk-associated palmitoylcarnitine.

In further studies it will be interesting to determine if the supplement can benefit patients with insulin resistance, both with regard to plasma TAG levels, mitochondrial function, and glucose tolerance. It will be of particular interest to compare the bioactivity of MOPL30 and TAG EPA and DHA supplements at lower doses of EPA and DHA. In a follow-up study, a double blind comparison to fish oil will be performed to identify possible PL-specific effects of MOPL30. Also, a rodent study is planned to examine bioaccretion of EPA and DHA into brain and other tissues.

## Conclusions

EPA and DHA-rich PC from herring roe was taken up by RBC during the two week intervention. Several parameters in blood were affected, including a reduction in TAG and NEFA, and an increase in HDL cholesterol, choline, and betaine. Further, there was improved glucose tolerance among the participants after two weeks. Based on the findings from this short-term pilot study with 2.4 g EPA + DHA per day, MOPL30 may provide significant effects on lipid status and glucose tolerance.

## Methods

### Study subjects

This intervention study was performed at the University of Bergen according to Good Clinical Practice Guidelines and the World Medical Association Declaration of Helsinki. The Regional Ethics Committee, REK vest, approved the protocol (REK vest, approval no. 2013/112), and informed consent was obtained from all the subjects.

Healthy young adults (21 women and one man) aged 21 to 26 years were recruited on a voluntary basis. Of the 27 adults asked, six were not included in the study due to unwillingness to take capsules and/or blood samples. Exclusion criteria were conditions requiring medication, pregnancy and diabetes type I or II. The participants were instructed not to make any major changes to their diet three weeks before, during, or four weeks after intervention, with the exception of avoiding using fish egg products like caviar. Participants were instructed to perform a three-day weighed food record within the two weeks before, as well as during the intervention. Results were analyzed by the program “Mat på Data” (http://www.matportalen.no/verktoy/mat_pa_data/), and mean dietary intake of nutrients of interest were calculated as percentage of total energy intake (energy %).

### Supplement and study design

MOPL30 is a capsulated herring roe PL supplement, where each capsule contains 511 mg total lipid, of which 30% are PL, with 56 mg EPA, 158 mg DHA, and 12 mg n-3 DPA. The participants received 11 capsules per day for 14 days, corresponding to a daily dose of 1738 mg DHA and 616 mg EPA. Four capsules were taken at breakfast and lunch and three capsules were taken at dinnertime. The last day of capsule intake (end), blood samples were drawn the next morning between 8 and 11 am after an overnight fast, an oral glucose tolerance test was performed (see description below), and capsules were divided between the remaining meals of the day. Blood samples were drawn the next morning after taking the last capsule between 8 and 11 am after an overnight fast, followed by an oral glucose tolerance test. Of the 21 participants, 14 were recruited for additional fasting blood samples one week (WO week one) and four weeks (WO week four) after the final day of supplement. One participant was excluded due to lack of fasting at WO week four. All blood samples were centrifuged and EDTA-plasma and serum was separated after a minimum of 15 minutes and maximum of 30 minutes at room temperature. Blood samples for isolation of RBC were drawn in EDTA tubes, centrifuged at 3000 rpm for 10 minutes, and plasma and interface removed. RBC were subsequently washed three times in PBS, with centrifugation and removal of buffy coat between each wash. All samples were aliquoted and stored at −80°C for further analysis.

### Oral glucose tolerance test

After an overnight fast, blood was drawn for measurement of fasting glucose and insulin as described above. In addition, a rapid analysis of blood glucose was performed using a FreeStyle Lite (Abbott Diabetes Care, Inc., Alameda, CA, USA). Glucose dissolved in water was ingested in no more than 5 minutes (300 ml 0.25 g/ml glucose with 5% lemon juice), and blood glucose was measured by FreeStyle Lite at 10, 30, 60 and 120 minutes after glucose ingestion. The area under the curve was calculated by Prism Graph-Pad Software (San Diego, CA, USA).

### Enzymatic analysis of blood parameters

Lipids were measured enzymatically in EDTA plasma on a Hitachi 917 system (Roche Diagnostics GmbH, Mannheim, Germany) using the triacylglycerol (GPO-PAP), cholesterol (CHOD-PAP), HDL-cholesterol plus and LDL-cholesterol plus kit from Roche Diagnostics, and the non-esterified fatty acid (NEFA FS) kit and the Phospholipids FS kit from DiaSys Diagnostic Systems GmbH (Holzheim, Germany). Glucose was measured in EDTA-plasma using the Gluco-quant Glucose/HK (GLU) kit from Roche Diagnostics. Insulin was measured using routine methods at the central laboratory at Haukeland University Hospital.

### Analysis of plasma total fatty acid composition, and plasma and RBC PC fatty acid composition

The total fatty acid composition in EDTA-plasma was analyzed as previously described [45]. For analysis of the plasma PC fatty acids, plasma total lipids were extracted based on Folch, then PC was separated from other lipids by HPLC using YMC diol-120-NP column, 250 mm × 4.6 mm ID, using hexane/acetone/methanol/chloroform (1/1/6/4) as the eluting solvent system. The column effluent was spilt to an evaporative light scattering detector for quantitation and to fraction collector for recovery. Fatty acids in the PC fraction were converted to their respective methyl esters then separated and quantified by capillary column GLC [46].

### Analysis of plasma and RBC lipid composition by ^1^H or ^31^P NMR

Lipids were extracted from 0.5 ml serum by Folch Solvent (1 ml each of CDCl3 MeOD and CsEDTA (0.2 M), pH 8). After centrifugation, the lower layer was analyzed at 600 MHz cQNP using a NMR spectrometer Avance III 600 (Bruker, Karlsruhe, D), magnetic flux density 14.1 Tesla, a QNP cryo probe, and automated sample changer Bruker B-ACS 120. Computer Intel Core2 Duo 2.4 GHz under MS Windows XP and Bruker TopSpin 2.1 was used for acquisition, while Bruker TopSpin 2.1 was used for processing [47-50].

### Plasma choline, betaine, carnitine and acylcarnitines

Plasma choline, betaine, free carnitine and its precursors: trimethyllysine and γ-butyrobetaine, as well as short-, medium-, and long-chain acylcarnitines, were analysed in plasma using LC/MS/MS as described previously [10]. Stable isotope dilution LC/MS/MS was used for quantification of choline and betaine. Choline and betaine were monitored in positive MRM MS mode using characteristic precursor-product ion transitions: *m/z* 76 → 58, *m/z* 104 → 60 and *m/z* 118 → 58, respectively. The internal standards, choline-trimethyl-d9 (d9-choline) and d11-betaine, were added to plasma samples before protein precipitation, and were similarly monitored in MRM mode at *m/z* 85 → 66, *m/z* 113 → 69 and *m/z* 129 → 66, respectively. Various concentrations of choline and betaine standards and a fixed amount of internal standards were spiked into 4% albumin (BSA) to prepare the calibration curves for quantification of plasma analytes.

### Statistical analysis

Data was analyzed using Prism Software (Graph-Pad Software). The results are shown as means with standard deviation (SD). D’Agostino & Pearson omnibus normality test was used to determine normal distribution. Paired *t*-test or Wilcoxon matched-pairs signed ranked test, for parametric data and non-parametric data, respectively, were performed to evaluate statistical differences between start and end samples, between end and WO week one, and between end and WO week four samples. P-values < 0.05 were considered significant.

## Competing interests

This work was partly supported by Arctic Nutrition AS, and at the time of the study, AB was an employee of Arctic Nutrition.

## Authors’ contributions

BB, ES, JG, AB, and RKB planned and designed the study. BB, ES and JG performed the study. PB performed the total plasma fatty acid composition assay. AS, BWKD, and SMI were responsible for the acylcarnitine analysis, NMR plasma lipid composition analysis, and the phosphatidyl choline fatty acid composition analysis, respectively. BB and AB performed statistical analysis, analysed the data, and BB wrote the manuscript. All authors critically revised the manuscript, and read and approved the final manuscript.
